# mHealth App for Pressure Ulcer Wound Assessment in Patients With Spinal Cord Injury: Clinical Validation Study

**DOI:** 10.2196/26443

**Published:** 2021-02-23

**Authors:** Ariane Do Khac, Claire Jourdan, Sylvain Fazilleau, Claire Palayer, Isabelle Laffont, Arnaud Dupeyron, Stéphane Verdun, Anthony Gelis

**Affiliations:** 1 Unité de Rééducation Neurologique Département de Médecine Physique et de Réadaptation Centre Hospitalo-Universitaire Nîmes France; 2 Département de Médecine Physique et de Réadaptation Centre Hospitalo-Universitaire Montpellier France; 3 Centre Mutualiste Neurologique Propara Montpellier France; 4 Délégation à la Recherche Clinique et à l'Innovation Groupement des Hôpitaux de l'Institut Catholique de Lille Lille France

**Keywords:** mobile app, wound, pressure ulcer, assessment, validity, reliability, app, wound, correlation, access, availability, reproducibility

## Abstract

**Background:**

Clinical evaluation of a pressure ulcer is based on quantitative and qualitative evaluation. In clinical practice, acetate tracing is the standard technique used to measure wound surface area; however, it is difficult to use in daily practice (because of material availability, data storage issues, and time needed to calculate the surface area). Planimetry techniques developed with mobile health (mHealth) apps can be used to overcome these difficulties.

**Objective:**

The goal of this study was to evaluate the metrological properties of a free-access mHealth app, called imitoMeasure, to assess pressure ulcers.

**Methods:**

This was a noninterventional, validation study. We included patients with spinal cord injury presenting with a pressure ulcer, regardless of its stage or location. We performed wound measurements with a ruler, and we performed acetate tracing using a transparent dressing with a wound measurement grid. Wound evaluation via the mHealth app was conducted twice by the main investigator and also by a coinvestigator to determine validity, intrarater reproducibility, and interrater reproducibility. Bland-Altman plots and intraclass correlation coefficients were used to compute the minimal detectable change percentage.

**Results:**

Overall, 61 different pressure ulcers were included. The validity, intrarater reproducibility, and interrater reproducibility of the mHealth app vs acetate tracing (considered the method of reference) were good, with intraclass correlation coefficients of 0.97 (95% CI 0.93-0.99), 0.99 (95% CI 0.98-0.99), and 0.98 (95% CI 0.96-0.99), respectively, and minimal detectable change percentages between 17% and 35%.

**Conclusions:**

The imitoMeasure app had good validity and reproducibility. It could be an alternative to standard wound assessment methods. Further studies on larger and more diverse wounds are needed.

**Trial Registration:**

ClinicalTrials.gov NCT04402398; http://clinicaltrials.gov/ct2/show/NCT04402398

## Introduction

Pressure ulcers are localized damage to the skin and underlying tissue resulting from long-term pressure or pressure in combination with shear or friction [1]. Despite major prevention efforts in hospitals or rehabilitation centers these past decades, pressure ulcers remain a major public health issue. The National Pressure Ulcer Advisory Panel (NPUAP) reports a prevalence of pressure ulcers of 2.3% to 28% in long-term facilities, 10% to 18% in intensive care units, and up to 6% in rehabilitation facilities [2]. Pressure ulcers are of concern in older adults, persons with neurological impairments (such as spinal cord injury), persons in intensive care units, and persons in palliative care.

Pressure ulcers have consequences on physical health (increased risk of other complications secondary to immobility, infectious risk, increased malnutrition) but also on mental health (isolation, boredom, depression) and social life (cessation of professional and social activities).

Pressure ulcer care management is based on managing general risk factors (nutrition, prevention, early mobilization) and on local cleansing care and wound dressing; sometimes, surgical debridement is necessary [3].

Care management requires rigorous and regular monitoring of the wound. Wound assessment includes qualitative evaluation (appearance of the wound, borders, perilesional skin, exudates [4]) and quantitative evaluation (volumetric or wound surface). For the latter, a method or reliable tool should facilitate reproducible evaluations from one assessor to the next and be responsive to wound changes, even minimal ones.

The standard method for wound measurement is acetate tracing. It is performed using a transparent acetate paper positioned over a graph dressing to manually calculate the area of the wound. This technique is not often used due to the hygiene risk (contact with the wound) and because it is time consuming. A more rapid estimation of the surface area can be performed with the Kundin method [5]; however, this method is based on the supposition that the wound has an elliptical shape, and thus becomes rather approximate when the wound shape is different. It is also possible to measure the wound using digital planimetry, a method in which the wound’s borders are delineated by the clinician using a computerized pen. For example, with the Visitrak system (Smith & Nephew plc), a transparent tracing of the wound is placed on a digital tablet, and its outline is retraced with a digital pen [6]. Its use remains quite rare. Finally, it is also possible to measure wound parameters with standard photography, which enables both qualitative and quantitative evaluation; the main inconvenience of this method is the need for secondary data processing of the image with computer software to obtain surface area (by counting the number of pixels) [5].

Smartphones and digital tablets are now routinely used in clinical practice. Most clinicians own a smartphone and use it for work to access information or to use mobile health (mHealth) apps [7]. This past decade, the development of mHealth apps, especially those for the quantitative evaluation of wounds, has greatly increased. Simply by taking a picture with a smartphone, apps such as MOWA (Healthpath) [8] or Swift Wound (Swift Medical) [9] can compute the dimensions and surface area of the wound. These mHealth apps have several advantages: simultaneous quantitative and qualitative wound evaluation, quick use, and no specific material requirements. The main limitations of these mHealth apps are their access (most require payment) and the lack of evidence regarding their validity for real wounds [10,11].

The objective of this work was to evaluate the metrological properties of a contactless digital planimetry app (imitoMeasure, Imito Ltd) for pressure ulcer measurement (width, length, surface area) in persons with spinal cord injury.

## Methods

### Study Design

We conducted a noninterventional study between May 2, 2019, and February 7, 2020, at the *Centre Mutualiste Neurologique Propara*, a rehabilitation center specializing in the care management of persons with spinal cord injury, to validate a contactless digital planimetry app (imitoMeasure, Imito Ltd). Each year, approximately 300 patients are seen as outpatients for pressure ulcer monitoring, and 45-50 patients are seen as inpatients for pressure ulcer surgery.

### Population and Procedure

Any patient seen for outpatient or inpatient pressure ulcer care management at the *Centre Mutualiste Neurologique Propara* during the study period was screened for inclusion. Inclusion criteria were (1) traumatic or nontraumatic spinal cord injury, regardless of time since injury, injury level, and complete or incomplete nature of the injury and (2) presenting with a Stage 2 to Stage 4 NPUAP pressure ulcer. Exclusion criteria were (1) age <18 years, (2) being an adult patient under legal guardianship, or (3) having a Stage 1 pressure ulcer (since the main objective of this app was to evaluate open wounds).

Data collection and pressure ulcer measurements were performed on the day of the patient’s regular consultation for wound evaluation and management. Routine care management of the patient was not modified. If the patient returned several times for the same pressure ulcer, only one measurement was included in the analysis; however, for a given patient, different pressure ulcers could be included.

Potential participants received oral and written information on the study; participating patients provided consent for data use and processing. The research protocol received authorization from the Montpellier University Hospital ethics committee (2019_IRB-MTP_06-02), and the study was registered at ClinicalTrials.gov (NCT04402398).

### Data Collected

Anthropometric data (height, weight) were collected along with time since injury, injury level, and American Spinal Injury Association Impairment Scale (AIS) score, which is the spinal cord injury scale of reference to assess severity [12].

### Wound Assessment

The duration and location of the wound were recorded. The length and width of the wound were measured with a ruler—the method that is most commonly used [13,14]. Length *L* was defined as the longest dimension, and width *W* was defined as the perpendicular measurement. The surface area *S* was evaluated in two ways: (1) using the Kundin formula [14] to give an approximation of the surface area by considering the wound as an ellipse, *S* = *L* × *W* × 0.785, and (2) performing acetate wound tracing [15] using Opsite Flexigrid (Smith & Nephew plc) dressings. The wound area on the acetate tracing was calculated manually by the main investigator by counting completely filled squares within the wound border and regrouping the partially full squares. This second evaluation method was considered as the technique of reference.

Wound length, width, and surface area were also measured with the imitoMeasure app [16]. The investigators used, according to their preference, an iOS (iPad mini, iPhone) or Android-based device.

**Figure 1:**
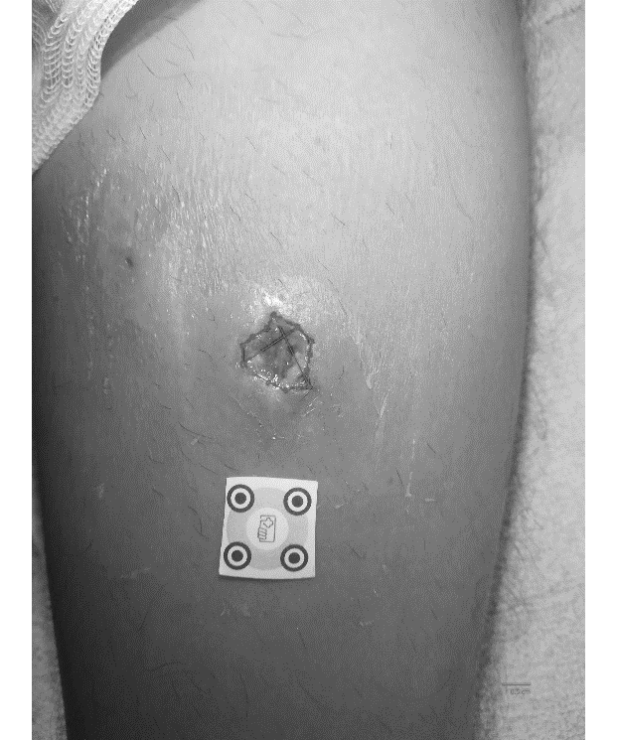
Image of the wound taken with the imitoMeasure app, with the calibration marker and wound borders manually delineated.

To use imitoMeasure, an adhesive calibration marker was placed next to the wound to calibrate the image. The calibration marker is freely accessible with the app and must be printed by the user. The picture was taken with the smartphone, using the app, then the wound borders were manually delineated on the screen (Figure 1). Length, width, and surface area measurements were then automatically calculated.

### Description of the Different Evaluations

The objective was to validate the use of the measurement tool in daily clinical practice; therefore, all health care professionals treating pressure ulcers at the *Centre Mutualiste Neurologique Propara* participated in the study. Overall, 12 evaluators, including 7 physicians and 5 nurses performed the assessments; all evaluators were trained to use the imitoMeasure app. For the first evaluation, the evaluator measured the length and width with a ruler, then delineated the wound borders on acetate paper; finally, measurements were taken with the imitoMeasure app. The second evaluator then performed new measurements with the imitoMeasure app (for the interrater reproducibility). The first evaluator then performed another evaluation with the app (intrarater reproducibility). Measures were anonymized and stored in an online medical database (REDCap, Vanderbilt University). The surface area was calculated with the Kundin formula automatically. Each evaluator entered data independently. To optimize the reproducibility of the surface area measurements, the wound tracing measurement was performed independently, by a single blinded investigator, based on the acetate tracings provided.

### Sample Size Calculation

In the absence of previously published clinical data on wound evaluations via the mobile app, we followed good practice guidelines for validation studies of measurement tools such as COSMIN criteria [17], considering that a minimum sample of 50 participants was adequate.

### Evaluation Criteria and Statistics

The main measure studied for validation was the wound surface area; the surface area measurement obtained via the imitoMeasure app was compared to each method. Measurements (length and width) obtained via the app were also compared to measurements obtained with the ruler. Measurements repeated the same day were used to evaluate inter- and intrarater reproducibility for imitoMeasure wound surface area assessment. Bland-Altman plots [18] were used for validity and reproducibility evaluations. Intraclass correlation coefficients (ICC) for the agreement parameters were computed (ICC 2.1 according to Shrout and Fleiss [19]). The 95% confidence interval of the ICC was calculated via bootstrapped distribution with 1000 replications. The ICC was considered good if >0.75 and excellent if >0.90 [20].

Additional analyses were conducted for reproducibility measures. The standard error of measurement was calculated from the components of the ICC variation [21], and the minimal detectable change (MDC) at 90% was obtained from the standard error. The MDC [22,23], also called smallest detectable change [21], represents the minimal detectable change between 2 measurements in order to be 90% certain that this change does not solely reflect measurement error [24]. If heteroscedasticity was observed from visual analysis of the Bland-Altman plots (ie, increased differences between the measures according to their mean values), the MDC was also expressed as a percentage, which could be interpreted as the minimal detectable change compared to the initial size of the pressure ulcer.

Subgroup analyses were conducted (validity and reproducibility) according to the location of the wound—either on flat (sacrum, flank, iliac crest, tibia, edge of the foot) or curved skin (ischial tuberosity, greater trochanter, calcaneus, occiput, malleolus). Statistical analyses were performed with R software (version 3.6.0 [25]; psy package [26] for ICC, boot package for confidence intervals). Excel (Microsoft Inc) software was used for graphs.

## Results

### Descriptive Analysis

A total of 61 pressure ulcers for 59 patients were studied (Table 1). Patients were mainly men, with thoracic spinal cord injury and AIS grade A. Most pressure ulcers were located on the ischial tuberosity. Their mean surface area was 7.7 cm^2^, ranging from 0.2 cm^2^ to 49.2 cm^2^ and a median at 4.7 cm^2^. The wound evaluations with the mobile app were performed using smartphones (Samsung S5, Samsung S8, iPhone 5S, iPhone 7, Huawei P8 Lite, Altice S70) or tablets (iPad mini 4, iPad Air).

**Table 1:** Patient and wound descriptive data.

Characteristic				Value, n (%)
**Patient (n=59)**				
	**Sex**			
		Male	52 (88)	
		Female	7 (12)	
	**Level of injury**			
		Cervical	7 (12)	
		Thoracic	47 (80)	
		Lumbar	5 (8)	
	**American Spinal Injury Association Impairment Scale**			
		Grade A	78 (81)	
		Grade B	6 (10)	
		Grade C	4 (7)	
		Grade D	1 (2)	
	**BMI^a^**			
		<18.5 kg/m^2^	3 (7)	
		18.5-25 kg/m^2^	23 (55)	
		>25 kg/m^2^	16 (38)	
**Wound characteristics (n=61)**				
	**Localization**			
		Sacrum	12 (20)	
		Ischial tuberosity	26 (43)	
		Trochanter	4 (6)	
		Heel	7 (11)	
		Other	12 (20)	
	**Age of wound^b^**			
		<2 weeks	7 (14)	
		2 weeks to 1 month	8 (16)	
		1 month to 6 months	17 (35)	
		6 months to 1 year	8 (16)	
		>1 year	9 (18)	

^a^n=42; data are missing from 17 patients.

^b^n=49; data are missing from 12 patient wounds.

### Validity of Wound Measurement via the imitoMeasure mHealth App

**Figure 2:**
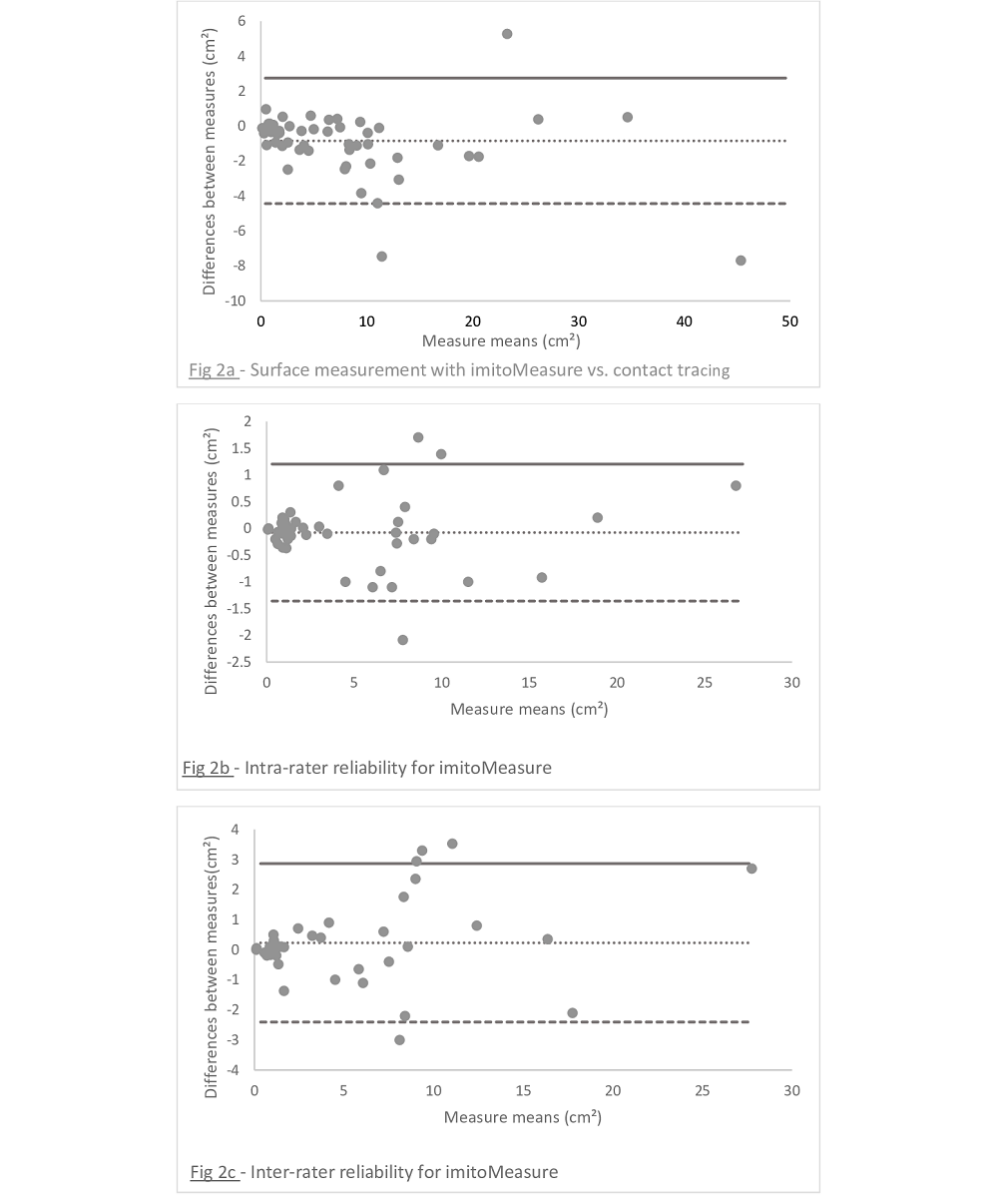
Bland-Altman plots: (a) validity (imitoMeasure app vs acetate tracing), (b) intrarater reproducibility, and (c) interrater reproducibility.

The comparison of wound surface area measurements with the imitoMeasure app and those from acetate tracing (the reference method) yielded an ICC of 0.97 (95% CI 0.93-0.99). The Bland-Altman plot for this comparison (Figure 2) showed a systematic bias close to zero (–0.8 cm^2^). Differences between measurements did not change with pressure ulcer size.

The comparison between measurements with the imitoMeasure and those using the Kundin method yielded an ICC of 0.96 (95% CI 0.92-0.97). For comparisons of length and width measurements with the imitoMeasure app and those with a ruler, ICCs ranged between 0.95 and 0.97.

### Reproducibility of Measures via the imitoMeasure App

For intra- and interrater reproducibility of wound surface area measurements, the Bland-Altman plots showed heteroscedasticity, with differences between increasing measures for larger pressure ulcers.

Intrarater reproducibility ICCs were greater than 0.98 both for dimensions (length and width) and surface area. MDC percentages varied between 15% and 19%. Interrater reproducibility ICCs were greater than 0.97, with MDC percentages ranging from 18% for length to 35% for the surface area (Table 2).

**Table 2:** imitoMeaure validity and reproducibility data.

Measure					ICC^a^ (95% CI)			SE			MDC^b^ cm or cm^2^ (%)^c^
**Validity**											
	Acetate tracing vs imitoMeasure (n=60)			0.97 (0.93-0.99)			N/A^d^			N/A	
	Kundin surface measure vs imitoMeasure (n=61)			0.96 (0.92-0.97)			N/A			N/A	
	Length ruler vs versus imitoMeasure (n=61)			0.97 (0.94-0.99)			N/A			N/A	
	Width ruler vs imitoMeasure (n=61)			0.95 (0.90-0.98)			N/A			N/A	
**Intrarater reproducibility**											
	Surface area (n=46)			0.99 (0.98-0.998)			0.45			1.05 (17)	
	Length (n=47)			0.99 (0.98-0.99)			0.21			0.50 (15)	
	Width (n=47)			0.98 (0.93-0.99)			0.16			0.38 (19)	
**Interrater reproducibility**											
		Surface area (n=44)	0.98 (0.96-0.99)			0.93			2.17 (35)		
		Length (n=44)	0.99 (0.97-0.99)			0.26			0.61 (18)		
		Width (n=44)	0.97 (0.94-0.99)			0.21			0.48 (24)		
**Subgroup analysis according to the wound location**											
	**Flat skin**										
		Validity (n=22)	0.98 (0.95-0.99)			N/A			N/A		
		Intrarater reproducibility (n=19)	0.99 (0.95-0.999)			0.47			1.10 (16)		
		Interrater reproducibility (n=16)	0.99 (0.98-0.997)			0.68			1.61 (23)		
	**Curved skin**										
		Validity (n=38)	0.97 (0.88-0.99)			N/A			N/A		
		Intrarater reproducibility (n=27)	0.99 (0.98-0.998)			0.43			1.01 (18)		
		Interrater reproducibility (n=28)	0.97 (0.93-0.98)			1.05			2.45 (43)		

^a^ICC: intraclass correlation coefficient.

^b^MDC: minimal detectable change.

^c^Percentage minimal detectable change in relation to the initial measurement.

^d^N/A: not applicable.

### Impact of the Wound Location

In subgroups, ICCs remained greater than 0.97, but only MDC values for interrater reproducibility were altered by measurement conditions, with a less reproducibility for pressure ulcer measurements on curved skin (MDC percentage 43%). MDC percentages for intrarater reproducibility were 16% and 18% for wounds on flat and curved skin, respectively.

## Discussion

### Principal Findings

This study is the first clinical evaluation that follows good practice guidelines for metrological quality [17] to measure pressure ulcers with an mHealth app. For surface area measurements performed via the imitoMeasure app, the validity and reproducibility based on the ICC was excellent (ICC>0.90). The evaluation of reproducibility with standard error and MDC, which is recommended [17,27] but scarcely used in the literature, yielded very satisfactory clinical results in a single rater, but unveiled some variability between raters for the same wound, potentially influenced by measurement conditions and wound location.

The interpretation of reproducibility requires methodological considerations. In fact, reproducibility encompasses 2 dimensions [21]. The so-called reproducibility is the measurement’s capacity to differ between 2 different individuals, well reflected by the ICC, which was excellent in our study. However, the ICC is intrinsically affected by interrater variability [21,24] and yields very high values when the sample is heterogeneous, which was the case in our study, with a wide range of pressure ulcer sizes. Thus, it is important to also evaluate the so-called agreement reproducibility, reflecting the similarities between 2 measurements and thus related to measurement error [21,27]. The evaluation of agreement, very rarely used in the literature [5], is now part of the quality criteria in tool validation studies [27], essential for situations wherein it is important to detect slight variations [21], such as wound monitoring. The agreement is sometimes assessed with the coefficient of variation [24,28] but more commonly with standard error and MDC because, as they are expressed in the measurement unit, they are easier to interpret by the clinician [21]. Because of ICC limitations, in our results, only standard error and MDC measurements highlighted the impact of changing rater and measure conditions. In previously conducted reproducibility studies [14] on wound measurement tools and techniques, the Pearson R was sometimes used, but it is less recommended than the ICC for reproducibility [27]. When the ICC was calculated for reproducibility, it ranged between 0.70 and 0.99 for traditional methods (acetate tracing, Kundin) [15,29], and it ranged between 0.96 and 0.99 for mHealth app methods [30,31]. Thus, our ICC values are excellent. A few studies gave results on agreement reproducibility, including one for acetate tracing with planimetry analysis (coefficient of variation 7% [32]) and one for a complex instrumental method, stereophotogrammetry (coefficient of variation 6.8% [33]). To our knowledge, no study has previously measured standard error or MDC. To allow for comparison, we calculated a posteriori coefficient of variations for wound surface area measured with the imitoMeasure app and obtained 7.4% for intrarater reproducibility and 15% for interrater reproducibility.

In our study, agreement reproducibility results showed less interrater reproducibility (in percentage MDC) for wounds on curved skin areas (ischial tuberosity, calcaneus, occiput, or malleolus). This is potentially related to planimetry analysis itself. The mHealth app considers that the wound is located on the same plane as the skin marker used to calibrate measures. The surface area measured is the actual projection of the wound onto this plane, thus the approximation value of the measurement widens if the skin is not flat, or if the marker is not positioned correctly. A first step to improve this would be to standardize measurements and have the rater to orient the skin marker to the wound’s main plane. Another solution would be to take a video of the wound with the smartphone and a calibration marker to reconstruct a 3D image of the wound surface area before measuring it. This solution would be similar to photogrammetric techniques, using different point of views to assess a wound on curved skin [34].

### Strengths

The mHealth app was evaluated in ecological conditions by nurses and physicians experienced in evaluating chronic wounds (experienced using acetate tracing, which was used as the reference). They were familiarized with the app quickly, without any prior training aside from the guidelines given to users by the company and used different mobile devices (iOS or Android-based smartphone or tablets).

Given how quickly one can be familiarized with the app, the time saved during wound assessment, and results from this first validation study, use of the mHealth app could be implemented in clinical practice, taking into account reproducibility limitations during interrater evaluations and for wounds on curved skin surfaces listed above.

### Limitations

A standard responsiveness to change study would require longitudinal follow-up [27] with several evaluations per patient, which was not within the scope of this study; however, the MDC assessment gave an indirect evaluation of the tool’s responsiveness to change since it allows the determination of the value above which we consider that the wound has changed clinically.

If analyses on the overall population had enough statistic power, subgroups analyses were performed on moderate (between 30 and 49) to small (<30) size samples [17], thus they should be interpreted with caution. Furthermore, reproducibility depended on the size of the wound (Figure 2), and in this study sample, there were few large pressure ulcers. Further validation studies on larger wounds could be useful.

### Perspectives for Clinical Practice

In clinical practice, use of the imitoMeasure app has the advantage of bypassing difficulties related to acetate tracing (such as availability of the material, issue of data storage, time to compute the surface area), and it is easily accessible since physicians regularly use their smartphones [35].

### Perspectives for Clinical Research

There is a need to evaluate standardization of this app’s use in clinical practice to improve interrater reproducibility and to make use of additional technological updates to improve wound measurement on curved skin surfaces. Further studies are needed to evaluate the validity of the mobile app for other clinical indications, such as venous or arterial ulcers, burns, erythematous skin eruptions, or allodynic areas in neuropathic pain. Furthermore, additional studies on pressure ulcers could include NPUAP stage 1 wounds—intact skin with nonblanchable erythema.

With the expansion of digital health in wound monitoring, there is a growing interest for photographic evaluations performed by patients or their families and sent via email to a specialist. A simple picture allows physicians to conduct an acceptable qualitative analysis of the wound, especially to evaluate the infectious risk [36]. Furthermore, most patients are able to send pictures of their wound via a secure email service to a physician, who can then conduct a qualitative clinical interpretation if the quality of the picture is sufficient [37]. The relevance of an mHealth app in the quantitative evaluation of wounds by patients at home to send to a remote physician remains to be defined and could be the topic of further study.

### Conclusion

Excellent metrological qualities of the mHealth app imitoMeasure were demonstrated when compared to evaluation methods used in daily practice, in the evaluation of pressure ulcers in persons with spinal cord injury. Further studies, on other types of wound, are needed.

## Abbreviations

AIS: American Spinal Injury Association Impairment Scale
ICC: intraclass correlation
MDC: minimal detectable change
mHealth: mobile health
NPUAP: National Pressure Ulcer Advisory Panel
